# Growth of Private Equity and Hospital Consolidation in Primary Care and Price Implications

**DOI:** 10.1001/jamahealthforum.2024.4935

**Published:** 2025-01-17

**Authors:** Yashaswini Singh, Nandita Radhakrishnan, Loren Adler, Christopher Whaley

**Affiliations:** 1 Department of Health Services, Policy, and Practice, Brown University School of Public Health, Providence, Rhode Island; 2 Brookings Institution, Washington, DC

## Abstract

**Importance:**

Consolidation of physician practices by hospitals and private equity (PE) firms has increased rapidly. This trend is of particular importance within primary care. Despite its significance, there is no systematic evidence on the emerging trends in ownership affiliation of primary care physicians (PCPs) and its association with prices paid for physician services.

**Objective:**

To describe trends in hospital affiliation and PE affiliation in primary care and examine variation in negotiated prices paid by commercial insurers to hospital-affiliated, PE-affiliated, and independent PCPs.

**Design, Setting, and Participants:**

Data from PitchBook and IQVIA were used to examine hospital and PE affiliation PCPs. PCPs and their affiliations were linked to novel cross-sectional Transparency in Coverage data. A total of 226.6 million negotiated prices were analyzed for evaluation and management office visits (*Current Procedural Terminology* codes 99202 to 99205 and 99212 to 99215) across 4 national insurers (Aetna, Blue Cross Blue Shield, Cigna, and United Healthcare). Linear regressions were used to examine the association between hospital-affiliated, PE-affiliated, and independent PCPs and cross-sectional prices paid for physician services, with fixed effects for service, state, and insurers. Data were collected from January to June 2024, and data were analyzed from July to October 2024.

**Main Outcomes and Measures:**

The proportion of PCPs that are affiliated with hospitals and PE from 2009 to 2022. Using cross-sectional data from 2022, negotiated prices paid to physicians (physician professional fee) for office visits.

**Results:**

A total of 198 097 PCPs were analyzed. PCPs affiliated with hospitals increased from 25.2% (28 216 of 111 793) in 2009 to 47.9% in 2022 (82 890 of 172 964). Over the same period, 1.5% (2483 of 172 964) of PCPs became affiliated with PE firms. Relative to independent PCPs, negotiated prices for office visits were $14.91 (95% CI, 8.92-27.64) or 10.7% (95% CI, 10.1-11.4) higher for hospital-affiliated PCPs (*P* < .001) and $9.56 (95% CI, 2.24-14.55) or 7.8% (95% CI, 4.7-10.8) higher for PE-affiliated PCPs (*P* < .001).

**Conclusions and Relevance:**

In this cross-sectional study, nearly one-half of all PCPs were affiliated with hospitals, while PE-affiliated PCPs were growing and concentrated in certain regional markets. Relative to PCPs in independent settings, hospital-affiliated PCPs and PE-affiliated PCPs had higher prices for the same services.

## Key Points

**Question:**

What are the trends in hospital and private equity (PE) affiliation of primary care physicians and their association with prices paid for physician services?

**Findings:**

In this cross-sectional study including 198 097 primary care physicians, in 2022, nearly half of all primary care physicians (PCPs) were hospital-affiliated, while PE-affiliated PCPs were concentrated in certain regional markets. Relative to independent PCPs, negotiated prices for office visits were 11% higher for hospital-affiliated and 8% higher for PE-affiliated PCPs.

**Meaning:**

An increasing share of PCPs are affiliated with hospitals and PE firms; relative to PCPs in independent settings, hospital-affiliated and PE-affiliated PCPs had higher prices for the same services.

## Teaser

This cross-sectional study describes trends in hospital and private equity affiliation in primary care and examines variation in negotiated prices paid by commercial insurers to hospital-affiliated, private equity–affiliated, and independent primary care physicians.

## Introduction

Physicians are increasingly moving away from solo and small practices toward larger organizations. More than three-fourths of physicians are employed by health systems or corporate entities.^1,2^ Primary care, in particular, has attracted substantial corporate consolidation from hospitals and private equity (PE) firms, in part due to fragmentation of independent clinicians, increased demand for coordinated care, and the potential to negotiate higher payment rates from commercial insurers.^3,4^ From 2010 to 2016, the share of primary care physicians (PCPs) affiliated with health systems increased from 28% to 44%.^5^ In recent years, primary care has also garnered substantial interest from PE firms that acquire physician practices to build market power with add-on acquisitions.^6,7,8^ While there is no systematic evidence that compares the involvement of PE in primary care with that by hospitals and health systems, emerging evidence on PE acquisitions of physician practices raises concerns about PE’s potential to alter practice patterns with implications for health care prices.^9,10,11,12^

Given underinvestments in primary care, low relative payments, and clinician burnout,^13^ corporate investors in primary care may present a unique opportunity to improve the financial health of primary care practices by negotiating higher payment rates from payers and making investments in health information technology to facilitate value-based care contracts.^14^ Indeed, one stated reason physicians sell their practices to hospitals or health systems and PE firms is the potential to negotiate higher prices from commercial insurers.^15^ A number of studies document that health system affiliation of physician practices increases prices paid by commercial insurers for physician services.^16,17,18,19^ At the same time, the recent growth of PE acquisitions of physician practices has also been associated with increased prices in certain procedural specialties, including dermatology,^11,12^ ophthalmology,^20,21,22,23^ and gastroenterology.^8,12^ As growing evidence on PE’s potential to increase health care prices emerges,^8,10,11,22,24^ it is unclear if these findings generalize to physician practices in primary care, which may have different business structures, service lines, and management practices.

Additional evidence on the growth in hospital and PE affiliation in primary care can shed light on recent changes in physician organization and help guide competition policy in health care markets. It may also help identify the sources of the wide variations in prices paid for physician services observed across geographic areas for similar services.^25,26^ Finally, some physicians consider employment in PE-affiliated practices to be an alternative to health system employment.^9^ Understanding the growth in PE in primary care within the broader context of consolidation by hospitals will facilitate an improved understanding of the changing landscape of the physician practice ecosystem.

Despite the importance of understanding the growth of hospital and PE affiliation in primary care and its association with health care prices, measuring this activity has been difficult given a lack of transparency. First, the lack of ownership transparency—ie, the absence of reporting and disclosure requirements for PE acquisitions—makes it difficult to identify the prevalence and geographic patterns in PE investments in physician practices. As a result, to our knowledge, there has been no systematic examination of trends in PE acquisitions in primary care. Second, until recently, the lack of price transparency made it difficult for patients, clinicians, researchers, and policymakers to understand sources of variation in prices across care settings. The recent implementation of the Transparency in Coverage (TiC) rules—requiring health insurers to disclose in-network negotiated rates for all services and contracted health care professionals—provides a novel opportunity to undertake this analysis.^27^

The objective of our study is 2-fold. First, we describe trends in the share of PCPs that are affiliated with hospitals or PE using longitudinal data on practice affiliation from 2009 to 2022, one of the longest study periods in the literature. Second, using cross-sectional TiC data from 2022, we examine how negotiated prices paid for physician services vary across ownership type.

## Methods

We constructed our analytical sample in multiple steps, first identifying hospital-affiliated and PE-affiliated PCPs, then linking this information to outcomes of interest constructed using TiC data. Data were collected from January to June 2024, and data were analyzed from July to October 2024; see the eMethods in Supplement 1 for additional background on data and methodology. The study was exempt from oversight by the Brown University Institutional Review Board. This study followed the Strengthening the Reporting of Observational Studies in Epidemiology (STROBE) reporting guideline for observational studies.

### Identifying Practice Affiliation

We obtained information on practice affiliation from the SK&A Office-Based Physicians Database from 2009 to 2019 provided by IQVIA, a commercial database of health care professionals, which provides a nearly complete sampling frame of US office-based physicians. SK&A data include clinician-level information (eg, age, location, specialty, clinician credentials, National Provider Identifier) and practice-level information, including hospital affiliation, and has been used in prior research to examine vertical integration of hospitals and health systems with physician practices.^28,29^ To identify PCPs, we limited our analysis to those with MDs and DOs with specialties listed as Family Practitioner, General Practitioner, and Internist.

We combined several data sources to identify PE affiliation of PCPs. First, we used proprietary data from PitchBook, a financial database that has been used by other studies examining PE in health care.^10,11,12,22,23,30^ We then manually verified and expanded this list using a combination of press releases and physician practice websites to identify standalone practice sites associated with each acquisition. Finally, to link PE acquisitions to SK&A data, we used probabilistic record linkage algorithms to link exact and nonexact records of practice names, addresses, and ownership information (eg, corporate parent identity) in the SK&A data to reported acquisitions. Overall, our linking strategy produced a match rate of 61% across all deal years.

To identify hospital affiliation of PCPs, we relied on attributes included in the SK&A data that list whether the practice is owned by or affiliated with a hospital or health system and, if it is owned, the owning entity. This approach is consistent with prior work examining changes in physician organization.^5,17,28^

### TiC Data

The TiC rules require health insurers to disclose in-network negotiated rates for all contracted services and clinicians.^27^ We obtained TiC data from Clarify Health through a data use agreement for rates negotiated in 2022 (eMethods in Supplement 1).

We focused on prices for evaluation and management office visits negotiated by 4 national insurers, including Aetna, Blue Cross Blue Shield, Cigna, and United Healthcare. These include negotiated professional fees for office visits for new patients (*Current Procedural Terminology* [CPT] codes 99202 to 99205) and established patients (*CPT* codes 99211 to 99215). Office visit codes are among the most commonly billed in the US.^31^ Data on *CPT* code 99201 were missing and excluded from our sample.

Our sample includes in-network rates submitted by commercial plans, where the disclosed prices represent negotiated rates rather than derived or per-diem rates or bundled payment arrangements. To remove outliers, we restricted our sample to prices between 75% and 1000% of Medicare rates. As TiC data postings can include zombie rates for physicians that do not perform services but are listed in an insurer’s pricing files, we limited our sample to physicians with billed claims for each relevant service code.^32^ Our sample included more than 226 million negotiated prices and 115 811 PCPs across ownership settings (eTable 1 in Supplement 1).

### Study Variables

We estimated the number of PCPs that are affiliated with hospitals and PE as a proportion of the total number of PCPs in the US in each year. Our primary outcome of interest is the physician professional fee negotiated by a given insurer for a specific service (*CPT* code) and physician (National Provider Identifier).

### Statistical Analysis

We first descriptively examined trends in hospital-affiliated and PE-affiliate PCPs by calculating, for each year in our sample, the number of PCPs that are affiliated with hospitals and PE as a proportion of the total number of PCPs. Using cross-sectional TiC data from 2022, we descriptively examined the weighted mean price for office visits across ownership type and the proportion of office visits that are low vs high complexity across ownership type. For new patient visits, we defined low-complexity visits to include *CPT* codes 99202 and 99203, and for established patient visits, we defined low-complexity visits to include *CPT* codes 99211 to 99213.

In regression-adjusted analyses, we examined the association between physician affiliation characteristics (independent variable) and negotiated prices for office visits (dependent variable). We used a fixed-effects regression with binary indicators for whether a physician is affiliated with a hospital or PE, weighted by total commercial service volume per physician, *CPT* code, and insurer-state fixed effects. Standard errors are clustered at the physician level. We used a Wald test to examine whether the difference in coefficients on hospital and PE affiliation is statistically significant.

In sensitivity analyses, we used an alternate approach from previous studies to identify hospital-affiliated physicians that relies on Medicare Data on Provider Practice and Specialty, the Medicare Provider Enrollment, Chain, and Ownership System, and IRS Form 990 tax forms to measure hospital-affiliated PCPs.^17,33,34^ In addition, we examined the robustness of our results to alternate regression specifications, including log-transformed outcome variables, unweighted regressions, and regression weights defined as insurers’ share of total service volume in a given state. We also estimated separate regressions for the top 2 services with the highest volume (*CPT* codes 99213 and 99214).

In secondary analysis, to examine price differentials between hospital-affiliated PCPs and all other PCPs, we used fixed-effects regression to evaluate the regression-adjusted difference between negotiated prices for hospital-affiliated PCPs relative to PCPs in all other settings (independent variable). We estimated this difference separately for each state by including interaction terms between binary indicators for each of the 50 states and a binary indicator for hospital affiliation.

Finally, we calculated commercial health care spending for office visits across ownership affiliation. We also estimated commercial spending for office visits under the assumption that prices for office visits would be the same across ownership affiliation and equal to the mean price at independent offices, and that the proportion of low-complexity visits across ownership settings would equal the proportion of low-complexity office visits at independent offices. In sensitivity analyses, we used median rather than mean prices.

Statistical tests were 2-tailed, and *P* values less than .05 were considered statistically significant. Data analyses were conducted using Stata SE version 17.0 (StataCorp).

## Results

**Figure 1.**
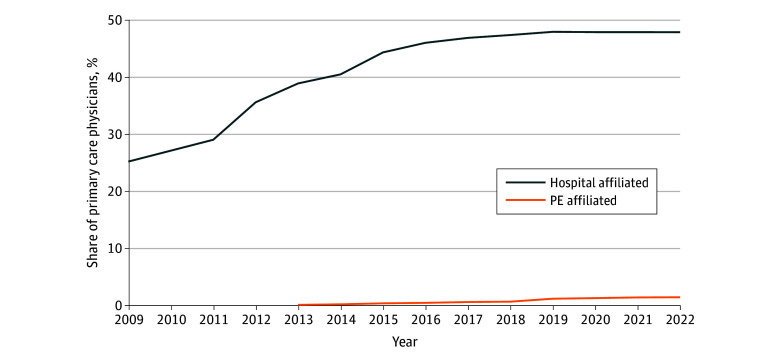
Trends in Hospital-Affiliated and Private Equity (PE)–Affiliated Primary Care Physicians From 2009 to 2022 Our calculation of data from PitchBook and IQVIA SK&A. The share of all primary care physicians that are affiliated with hospitals and PE is depicted. The share of PE-acquired physicians was less than 0.1% before 2013 and is not depicted.

A total of 198 097 PCPs were analyzed. The share of hospital-affiliated PCPs increased from 25.2% (28 216 of 111 793) in 2009 to 47.9% (82 890 of 172 964) in 2022 (Figure 1). Over the same period, the share of PE-affiliated PCPs increased to 1.5% by 2022 (2483 of 172 964) (eFigure 1 in Supplement 1).

**Figure 2.**
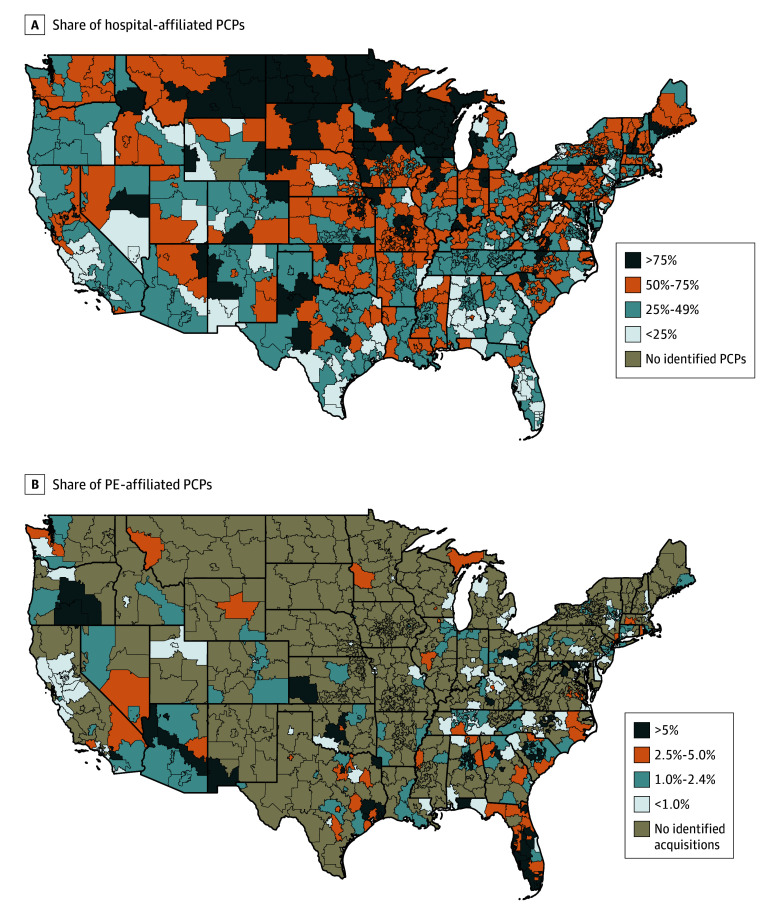
Geographic Variation in Hospital-Affiliated and Private Equity (PE)–Affiliated Primary Care Physicians (PCPs) in 2022 Estimates are based on our calculations of data from PitchBook and IQVIA SK&A. The unit of analysis is the 3-digit zip code.

Hospital-affiliated and PE-affiliated PCPs were concentrated in certain geographic markets (Figure 2). The states with the highest proportion of PCPs in PE-acquired practices were Florida (6.3% [728 of 11 489]) and Texas (3.4% [405 of 11 978]), while the states with the highest portion of hospital-affiliated PCPs were North Dakota (86.2% [356 of 413]) and Wisconsin (83.2% [2770 of 3329]). In general, states with high hospital affiliation rates tended to have low PE affiliation rates.

**Figure 3.**
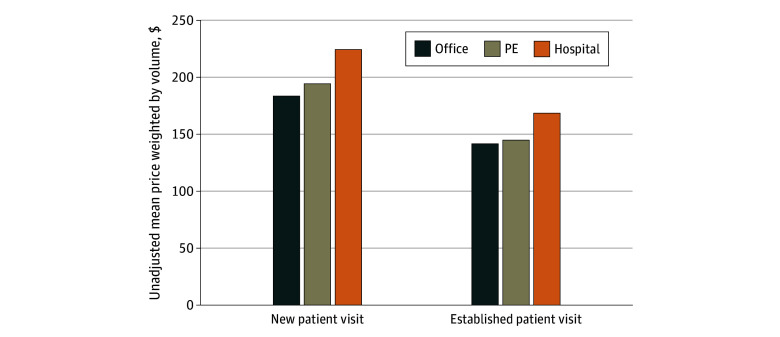
Unadjusted Mean Prices for Office Visits Across Ownership Type in 2022 Our analysis of PitchBook, IQVIA SK&A, and Transparency in Coverage data. This figure summarizes the unadjusted weighted mean price across 226.6 million prices for office visits with primary care physicians using cross-sectional Transparency in Coverage data from 2022. New patient visit includes *Current Procedural Terminology* codes 99202 to 99205, and established patient visit includes *Current Procedural Terminology* codes 99211 to 99215. PE indicates private equity.

Figure 3 summarizes the variation in weighted mean negotiated prices for office visits at hospital-affiliated, PE-affiliated, and independent PCPs. Among new patient visits, the unadjusted weighted mean (SE) negotiated price was highest for hospital-affiliated PCPs at $180 (0.14), followed by $155 (1.07) at PE-affiliated and $147 (0.10) at independent PCPs. Similarly, among established patient visits, mean (SE) unadjusted prices were $135 (0.08) for hospital-affiliated PCPs, $116 (0.68) for PE-affiliated PCPs, and $113 (0.07) for independent PCPs. Cross-sectional variation in negotiated prices across hospital-affiliated, PE-affiliated, and independent PCPs existed even within specific services (eFigure 2 in Supplement 1) and across insurers (eFigure 3 in Supplement 1).

Table 1 summarizes regression-adjusted variation in prices negotiated by hospital-affiliated, PE-affiliated, and independent PCPs. Negotiated prices for office visits were $14.91 (95% CI, 8.92-27.64) or 10.7% (95% CI, 10.1-11.4) greater for hospital-affiliated PCPs relative to independent PCPs (*P* < .001) (eTable 6 in Supplement 1), with wide variation across states (eFigures 4 and 5 in Supplement 1). Office visit prices for PE-affiliated PCP practices were $9.56 (95% CI, 2.24-14.55) or 7.8% (95% CI, 4.7-10.8) greater than prices for independent PCPs (*P* < .001). The *P* value for a Wald test that examines the null hypothesis the relative difference between hospital-affiliated and independent prices (ie, $14.91) and PE-affiliated and independent prices (ie, $9.56) are not different was .006, indicating a statistically significant difference between prices for office visits at hospital-affiliated vs PE-affiliated settings.

**Table 1.** Association Between Commercial Prices for Office Visits and Primary Care Affiliation, 2022^a^

Variable	Coefficient (SE; 95% CI)	*P* value
Ownership		
Independent office	1 [Reference]	NA
Private equity	9.56 (1.95; 2.24 to 14.55)	<.001
Hospital	14.91 (0.41; 8.92 to 27.64)	<.001
*CPT* code		
99203 (New patient office visit, 30-44 min)	40.41 (0.85; 40.74 to 52.33)	<.001
99204 (New patient office visit, 45-59 min)	110.20 (1.02; 106.65 to 134.10)	<.001
99205 (New patient office visit, >60 min)	183.46 (0.01; 175.79 to 209.85)	<.001
99212-99215 (Established patient office or other outpatient visit)	−63.27 (0.02; −74.31 to −61.27)	<.001
99212 (Established patient office or other outpatient visit, 10-19 min)	−31.88 (0.03; −36.07 to −30.35)	<.001
99213 (Established patient office or other outpatient visit, 20-29 min)	7.07 (0.02; 6.44 to 13.38)	<.001
99214 (Established patient office or other outpatient visit, 30-39 min)	52.87 (0.02; 50.51 to 66.58)	<.001
99215 (Established patient office or other outpatient visit, greater than 40 min)	115.66 (0.02; 106.80 to 138.01)	<.001
Constant	88.30 (0.84; 65.57 to 118.33)	<.001

Abbreviations: *CPT*, *Current Procedural Terminology*; NA, not applicable.

^a^ Table 1 summarizes regression coefficients obtained from a fixed-effects regression with physician professional fees as the dependent variable and binary indicators for ownership type (affiliated with a hospital or private equity) as independent variables. The reference category is primary care physician practices under independent ownership. Regressions include fixed effects for service (*CPT* code) and insurer-state (absorbed) and are weighted by the total commercial service volume for a given National Provider Identifier. SEs are clustered at the level of the physician. The *P* value for a Wald test of the null hypothesis that the coefficients on hospital and private equity affiliation are equal is .006.

Our finding that prices for office visits were highest at hospital-affiliated PCPs is consistent when using alternate data sources to identify hospital-affiliated PCPs (eTables 2 and 3 in Supplement 1), unweighted regressions (eTables 4 and 5 in Supplement 1), log-transformed outcomes (eTable 6 in Supplement 1), and for select services (eTable 9 in Supplement 1). In regressions that use insurer’s share of service volume, results for PE were qualitatively similar but not statistically significant (eTables 7 and 8 in Supplement 1).

Finally, we estimated that commercial spending on office visits exceeded $10.8 billion dollars at primary care practices affiliated with hospitals (Table 2). Under different assumptions about prices and service mix (eFigure 6 in Supplement 1), commercial spending on office visits at hospital-affiliated primary care settings would decrease by approximately $1.5 to $1.8 billion dollars or 15% to 18%. In sensitivity analyses using median prices, we found similar results (eTable 10 in Supplement 1).

**Table 2.** Changes in Health Care Spending Under Different Assumptions^a^

Model	Hospital affiliated		Private equity affiliated	
	Spending, $	Decrease in spending, %	Spending, $	Decrease in spending, %
Actual spending	10 895 326 641	NA	147 021 977	NA
Counterfactual assumption A: using mean price at independent offices	9 353 190 230	15	147 760 670	NA
Counterfactual assumption B: using mean price and service complexity at independent offices	9 040 655 594	18	143 179 866	NA

Abbreviation: NA, not applicable.

^a^ Actual spending is calculated as the sum of the product of mean negotiated price and service volume for each *Current Procedural Terminology* code. In counterfactual assumption A, we calculate health care spending using mean negotiated prices at independent settings and total service volume at hospital-affiliated and private equity–affiliated practices. In counterfactual assumption B, we calculate health care spending using mean negotiated prices and service-mix at independent settings.

## Discussion

The growing acquisition of physician practices by hospitals and health systems and PE firms is one of the most important recent trends in the organization of physician practices in the US. Our study provides comprehensive estimates of changes in ownership affiliation of PCPs over one of the longest study periods in the literature. Our results demonstrate that vertical integration of physician practices with health systems is still the dominant trend in primary care, with nearly one-half of all PCPs affiliated with hospitals. Although nationwide PE penetration in primary care is relatively low, PE investments in primary care have emerged as a notable trend in certain local and regional markets.

Our finding that prices are higher at hospital-affiliated settings relative to independent settings is consistent with prior research, which has found differences in commercial insurer payment rates for identical outpatient services delivered in physicians’ offices and hospital-affiliated settings.^26^ Using data from 2007 to 2013, Capps et al^16^ found that hospital acquisitions of physician practices increased prices for physician services by 14% relative to independent practices. Nearly a decade later, our cross-sectional results using new sources of price transparency data remain consistent with this finding. Given that price differences across care settings are more likely to be attributable to market factors than quality,^35,36,37^ if a desired policy goal is to reduce health care spending, hospital-physician vertical integration may deserve similar levels of policy attention as is currently received by PE.

Our finding that negotiated prices for office visits are higher for PE-affiliated PCPs relative to independent offices is consistent with growing evidence on the association of PE acquisitions and higher health care prices.^10,11,12^ While our findings are consistent with PE’s strategy of add-on consolidation that may increase prices paid by commercial insurers, given our cross-sectional data, we are unable to rule out another plausible explanation that higher prices in PE-affiliated practices reflect the strategic selection of high-priced platform practices as acquisition targets by PE firms (as alleged in the Federal Trade Commission’s lawsuit against a PE-backed anesthesia platform).^38^ On the other hand, given the recency of PE affiliation of PCPs relative to hospital affiliation, it is possible that PE might result in even higher prices than those currently observed as firms continue to embark on roll-up consolidation of smaller practices. Taken together, our study highlights the need for greater monitoring and transparency of corporate consolidation of physician practices, including by PE firms.

To our knowledge, this is the first known analysis to compare commercial prices across hospital-affiliated, PE-affiliated, and independent PCPs. Evidence suggests that relative market power between commercial insurers and physician organizations drives negotiated prices.^39,40^ The ability for hospital-affiliated and PE-affiliated practices to negotiate higher professional fees relative to independent physician practices highlights one significant reason that physician practices continue to be attracted to corporate ownership. In particular, given low levels of investment in primary care^41^ and concerns surrounding clinician burnout,^42^ corporate investors in primary care may present a unique opportunity to reduce clinician burnout by facilitating administrative support^14^ and investments in the use of technology into the primary care workflow.^43,44,45^ An important question is whether higher prices paid to hospital-affiliated and PE-affiliated practices also benefits patients and the clinical workforce. Prior research has shown that higher prices from vertical integration of physician practices with health systems do not accrue to physicians in the form of greater income.^28^ Thus, a key question that remains unanswered is whether revenues from higher prices negotiated by hospital-affiliated and PE-affiliated practices result in improved outcomes for patients and/or clinicians.

### Limitations

Our study has limitations. First, some PE acquisitions may have been missed. Second, use of secondary data from SK&A may include measurement error, although our results are consistent using alternate approaches to identifying hospital affiliation. Third, our analysis of negotiated prices is cross-sectional, which precludes any determination of causality. Relatedly, we are unable to examine potential mechanisms that explain variation in prices, including the selection of higher-priced practices as acquisition targets, increased market power from hospital acquisitions, or countervailing bargaining power of insurers. Fourth, hospital-affiliated, PE-affiliated, and independent practices may differ in unobserved ways that influence prices, including quality of care and patient composition. Fifth, our categorization of independent settings includes heterogeneous entities, for example, PCPs affiliated with retail companies and payers. Relatedly, hospital-affiliated physicians include heterogeneous organizational forms, including hospitals that are affiliated with PE. Our findings may not be generalizable to other payers or service categories. In particular, PE might drive additional effects in the Medicare Advantage population under capitated payment models. Finally, while we documented variation in prices based on physician affiliation, we were unable to track what share, if any, of higher prices is received by physicians in the form of compensation vs potentially captured by hospitals or PE firms. While increased market share enables better negotiation with insurers, it can also introduce monopsony power in wage negotiations.^46^ Existing research shows hospital-employed physicians do not have higher pay than independent physicians.^28^

## Conclusions

In this cross-sectional study, nearly one-half of all PCPs were affiliated with hospitals, and PE is emerging as a new form of corporate affiliation in primary care. Hospital-affiliated and PE-affiliated PCPs negotiated higher prices for evaluation and management visits relative to independent PCPs. Ownership and price transparency can uncover sources of price variation that will be important for policymakers to monitor.

## Notes

Supplement 1. **eMethods.** Background on Data and Methodology **eTable 1.** Number of Observations Across Ownership Type, by CPT Code **eFigure 1.** Share of Physicians in PE-Acquired Practices, 2009-2022 **eFigure 2.** Unadjusted Prices for Office Visits Across Ownership Type, by CPT Code **eFigure 3.** Unadjusted Prices for Office Visits by Ownership Type, by Insurer **eFigure 4.** Geographic Variation in Prices for Office Visits With Hospital-Affiliated Primary Physicians Relative to Other Outpatient Settings **eFigure 5.** Ranking of States With the Highest Price Differential Comparing Hospital-Affiliated Prices to All Other Prices **eTable 2.** Adjusted Variation in Commercial Prices for Office Visits by Ownership Type (Dollars, Alternate Data Source to Identify Hospital-Affiliated Physicians) **eTable 3.** Adjusted Variation in Commercial Prices for Office Visits by Ownership Type (Log-Transformed, Alternate Data Source to Identify Hospital-Affiliated Physicians) **eTable 4.** Adjusted Variation in Commercial Prices for Office Visits by Ownership Type (Dollars, No Regression Weights) **eTable 5.** Adjusted Variation in Commercial Prices for Office Visits by Ownership Type (Log-Transformed, No Regression Weights) **eTable 6.** Adjusted Variation in Commercial Prices for Office Visits by Ownership Type (Log-Transformed, Regression Weights Defined as Total Commercial Volume for a Provider) **eTable 7.** Adjusted Variation in Commercial Prices for Office Visits by Ownership Type (Rate, Regression Weights Defined as Insurer Share) **eTable 8.** Adjusted Variation in Commercial Prices for Office Visits by Ownership Type (Log-Transformed, Regression Weights Defined as Insurer Share) **eTable 9.** Adjusted Variation in Commercial Prices for Office Visits by Ownership Type (Dollars and Log-Transformed, Select CPT Codes) **eFigure 6.** Proportion of Low vs High Complexity Visits Across Ownership Type **eTable 10.** Changes in Health Care Spending Under Different Assumptions

Supplement 2. Data Sharing Statement
