# Supplementary material for: Growth of Private Equity and Hospital Consolidation in Primary Care and Price Implications
Source: JAMA Health Forum. 2025 Jan 17;6(1):e244935. doi: 10.1001/jamahealthforum.2024.4935 (PMC11742525; doi:10.1001/jamahealthforum.2024.4935)
Supplement: Supplement 1. — eMethods. Background on Data and Methodology eTable 1. Number of Observations Across Ownership Type, by CPT Code eFigure 1. Share of Physicians in PE-Acquired Practices, 2009-2022 eFigure 2. Unadjusted Prices for Office Visits Across Ownership Type, by CPT Code eFigure 3. Unadjusted Prices for Office Visits by Ownership Type, by Insurer eFigure 4. Geographic Variation in Prices for Office Visits With Hospital-Affiliated Primary Physicians Relative to Other Outpatient Settings eFigure 5. Ranking of States With the Highest Price Differential Comparing Hospital-Affiliated Prices to All Other Prices eTable 2. Adjusted Variation in Commercial Prices for Office Visits by Ownership Type (Dollars, Alternate Data Source to Identify Hospital-Affiliated Physicians) eTable 3. Adjusted Variation in Commercial Prices for Office Visits by Ownership Type (Log-Transformed, Alternate Data Source to Identify Hospital-Affiliated Physicians) eTable 4. Adjusted Variation in Commercial Prices for Office Visits by Ownership Type (Dollars, No Regression Weights) eTable 5. Adjusted Variation in Commercial Prices for Office Visits by Ownership Type (Log-Transformed, No Regression Weights) eTable 6. Adjusted Variation in Commercial Prices for Office Visits by Ownership Type (Log-Transformed, Regression Weights Defined as Total Commercial Volume for a Provider) eTable 7. Adjusted Variation in Commercial Prices for Office Visits by Ownership Type (Rate, Regression Weights Defined as Insurer Share) eTable 8. Adjusted Variation in Commercial Prices for Office Visits by Ownership Type (Log-Transformed, Regression Weights Defined as Insurer Share) eTable 9. Adjusted Variation in Commercial Prices for Office Visits by Ownership Type (Dollars and Log-Transformed, Select CPT Codes) eFigure 6. Proportion of Low vs High Complexity Visits Across Ownership Type eTable 10. Changes in Health Care Spending Under Different Assumptions [file jamahealthforum-e244935-s001.pdf]

Supplemental Online Content

Singh Y, Radhakrishnan N, Adler L, Whaley C. Growth of private equity and hospital consolidation in primary care and price implications. *JAMA Health Forum.* 2025;6(1):e244935. doi:10.1001/jamahealthforum.2024.4935

eMethods. Background on Data and Methodology

eTable 1. Number of Observations Across Ownership Type, by CPT Code

eFigure 1. Share of Physicians in PE-Acquired Practices, 2009-2022

eFigure 2. Unadjusted Prices for Office Visits Across Ownership Type, by CPT Code

eFigure 3. Unadjusted Prices for Office Visits by Ownership Type, by Insurer

eFigure 4. Geographic Variation in Prices for Office Visits With Hospital-Affiliated Primary Physicians Relative to Other Outpatient Settings

eFigure 5. Ranking of States With the Highest Price Differential Comparing Hospital-Affiliated Prices to All Other Prices

eTable 2. Adjusted Variation in Commercial Prices for Office Visits by Ownership Type (Dollars, Alternate Data Source to Identify Hospital-Affiliated Physicians)

eTable 3. Adjusted Variation in Commercial Prices for Office Visits by Ownership Type (Log-Transformed, Alternate Data Source to Identify Hospital-Affiliated Physicians)

eTable 4. Adjusted Variation in Commercial Prices for Office Visits by Ownership Type (Dollars, No Regression Weights)

eTable 5. Adjusted Variation in Commercial Prices for Office Visits by Ownership Type (Log-Transformed, No Regression Weights)

eTable 6. Adjusted Variation in Commercial Prices for Office Visits by Ownership Type (Log-Transformed, Regression Weights Defined as Total Commercial Volume for a Provider)

eTable 7. Adjusted Variation in Commercial Prices for Office Visits by Ownership Type (Rate, Regression Weights Defined as Insurer Share)

eTable 8. Adjusted Variation in Commercial Prices for Office Visits by Ownership Type (Log-Transformed, Regression Weights Defined as Insurer Share)

eTable 9. Adjusted Variation in Commercial Prices for Office Visits by Ownership Type (Dollars and Log-Transformed, Select CPT Codes)

eFigure 6. Proportion of Low vs High Complexity Visits Across Ownership Type

eTable 10. Changes in Health Care Spending Under Different Assumptions

This supplementary material has been provided by the authors to give readers additional information about their work.

© 2025 Singh Y et al. *JAMA Health Forum*.

eMethods. Background on Data and Methodology

To conduct our analyses, we constructed our dataset in multiple steps. To summarize the process, we 1) identified PE acquisitions in primary care using Pitchbook data and manual review of public sources, 2) identified physicians associated with each practice using datasets from IQVIA, including hospital-affiliated, PE-affiliated, and independent practices, 3) linked physicians and their ownership affiliated to outcomes of interest constructed using Transparency-in-Coverage (TiC) data.

Step 1: Identify PE Acquisitions

Our primary source of data on private equity transactions is a proprietary list of acquisitions in the "Clinics and outpatient services" sector compiled by Pitchbook Inc., a financial database that tracks mergers and acquisitions across industries and has been used by other studies examining PE in health care. Given that there is no single data source that tracks the complete universe of PE acquisitions of physician practices, a limitation of this data is that it might under report some PE acquisitions. To account for this, we supplemented Pitchbook data with manual searches described below.

Information on private equity (PE) acquisition of primary care practices from 2015 to 2022 was derived from Pitchbook. Acquisition data from Pitchbook included the name and a description of the acquired practice, city and state of the acquired facilities, a description of the deal, and the announcement date of the deal. We supplement the Pitchbook data in two ways. First, we conduct additional Internet searches that yielded an additional number of acquisitions not reported by Pitchbook. Second, we conduct additional searches to obtain a list of merger and acquisition deals from Mergr and CBInsights, market intelligence firms that track the healthcare sector. This process allowed us to manually verify and expand our list of acquisitions using a combination of press releases, industry reports, and physician practice websites. As a result, we were able to identify standalone practice sites associated with each practice acquisition, as well as to account for changes in practice names.

Step 2: Identifying physicians affiliated with practices

The SK&A Office Based Physicians database ("SK&A") includes data on physicians in office-based (outpatient) settings and their affiliated locations, including clinician-level information (e.g., age, location, specialty, clinician credentials, National Provider Identifier (NPIs)) and practice-level information, including ownership and corporate affiliations, on 9.7 million health professionals in the U.S. Previous research has found estimates of physician counts and affiliations obtained using IQVIA data to be comparable to those reported in the AMA Physician Masterfile data. This is also consistent with prior research that has demonstrated concordance of IQVIA data with publicly available data for office-based specialties.[1,2] A key advantage of these datasets is that they list distinct office sites belonging to a particular practice, allowing for more precise identification of practice locations acquired by PE.

© 2025 Singh Y et al. *JAMA Health Forum*.

To identify individual physicians (MDs and DOs) affiliated with both acquired and non-acquired practices, we merged acquisition data from Step 1 (including names and addresses of physician practice sites acquired by PE1) to SK&A data. To do this, we used probabilistic record linkage algorithms to link exact and non-exact records of practice names, addresses, and ownership information (e.g., parent organization). For any unmatched deals, we manually matched acquisitions to using publicly available information to verify practice locations and potential name changes for acquired practices. Overall, our linking strategy produced a match rate of 61% across all deal years, consistent with match rates in other studies examining PE in physician practices.[3]

To identify hospital-affiliated physicians, we relied on ownership attributes included in the SK&A data that list whether the practice is owned by a hospital or health system and, if it is owned, the owning entity. This approach is consistent with prior work examining changes in physician organization.[4–6]

As a sensitivity test to increase the reliability of the SK&A data, we used an alternate approach to identify hospital-affiliated physicians (eTable 2 and 3). This alternate approach uses relies on the Medicare Data on Provider Practice and Specialty (MD-PPAS), algorithms developed by the RAND Center of Excellence on Health System Performance, data from the Medicare Provider Enrollment, Chain, and Ownership System (PECOS) and IRS Form 990 tax forms to measure hospital-affiliated PCPs. This approach has been used in several published studies on vertical integration.[5,7,8] As we show in eTables 2 and 3, results from a sensitivity analysis using this alternate methodology to identify hospital-affiliated physicians is consistent with our main specification.

Step 3: Construct outcomes of interest using TiC data

We examine variation in cross-sectional prices by linking data from Step 1 and Step 2 above to the newly released “Transparency-in-Coverage” (TiC) data on insurer prices from 2022. The 2020 Transparency in Coverage (TiC) Executive Order requires insurance companies to post negotiated rates for all commercially insured contracts. For all insurers, these data contain price lists of procedure- and provider-specific prices.

Although released in July 2021, these data have not been widely used, largely due to data structure and complexity. Nevertheless, emerging research on the accuracy of the TiC data has found high-level of concordance among TiC data, hospital pricing data, and Marketscan commercial claims.[9,10] For example, a recent study found that insurer-disclosed prices in the TiC data have a correlation coefficient of 0.975 compared to prices disclosed by hospitals, with 77.4% prices matching to the penny, and 84.4% prices within 10%.[10]

Recently, a small number of private entities specializing in Transparency in Coverage have arisen to aggregate and clean this data, providing it to the business community as well as academic researchers. To analyze the TiC data, we use data from one such third-party source, Clarify Health, under a data use agreement.

Clarify Health aggregates TiC data from monthly updates of insurer TiC data postings. An existing limitation of many TiC data postings are the inclusion of “zombie rates” for providers that do not perform services but are listed in an insurer’s pricing files.[11] As a result, many reported observations reflect price observations for providers unlikely to perform a procedure. To address this data challenge, Clarify links TiC data to a 100 percent sample of Medicare fee-for-services (FFS) enrollees and commercial insurance claims data. Collectively, these claims data cover approximately 270 million people. To exclude insurer-provider combinations that are rarely observed, we limit observations to TiC prices for providers with billed claims for each relevant service code.

We link data on physicians and their practice affiliation (hospital-affiliated, PE-affiliated, and independent) to TiC data using physician NPIs. Overall, approx. ~59% of NPIs in the SK&A data were found in the TiC data, with no significant differences in match rates across affiliation types.

We limited our sample to prices from the four national insurers (Blue Cross Blue Shield, UnitedHealth Group, Cigna, Aetna). Humana was excluded as it did not have posted rates for commercial plans for selected CPT codes. Clarify Health aggregates data across plan types (PPO, HMO) for a given insurer to calculate a weighted average negotiated price across all plan types. We use this weighted average price in our analysis. Thus, if an insurer has enrollees in a PPO plan with a PPO price and enrollees in an HMO with an HMO price, the price used in our analyses would use a weighted average of those two prices as provided by Clarify.

In regression analyses, our primary outcome is the physician professional fee negotiated by a given insurer-physician pair for office visits for new patients (Current Procedural Terminology [CPT] code 99202-99205) and established patients (CPT codes 99211-99215). While office visits may generate a facility-based fee for services provided in hospital-affiliated settings, to allow for an apples-to-apples comparison, we examine only the physician professional fee across ownership types.

References

1. Cohen GR, Jones DJ, Heeringa J, et al. Leveraging diverse data sources to identify and describe US health care delivery systems. *eGEMs*. 2017;5(3).

2. Valdez S, Jacobson M. Assessing the Quality of SK&A’s Office-Based Physician Database for Identifying Oncologists. *Med Care Res Rev MCRR*. 2022;79(2):317-327. doi:10.1177/10775587211013628

3. Singh Y, Song Z, Polsky D, Bruch JD, Zhu JM. Association of Private Equity Acquisition of Physician Practices With Changes in Health Care Spending and Utilization. *JAMA Health Forum*. 2022;3(9).

© 2025 Singh Y et al. *JAMA Health Forum*.

4. Fulton BD. Health Care Market Concentration Trends In The United States: Evidence And Policy Responses. *Health Aff (Millwood)*. 2017;36(9):1530-1538. doi:10.1377/hlthaff.2017.0556

5. Whaley CM, Zhao X, Richards M, Damberg CL. Higher Medicare Spending On Imaging And Lab Services After Primary Care Physician Group Vertical Integration. *Health Aff (Millwood)*. 2021;40(5):702-709.

6. Whaley CM, Arnold DR, Gross N, Jena AB. Physician Compensation In Physician-Owned And Hospital-Owned Practices. *Health Aff Proj Hope*. 2021;40(12):1865-1874. doi:10.1377/hlthaff.2021.01007

7. Levin JS, Komanduri S, Whaley CM. Association Between Hospital-Physician Vertical Integration and Medication Adherence Rates. *Health Serv Res 2022 Doi 1011111475-677314090*. Published online October 28, 2022. Accessed October 24, 2024. https://www.rand.org/pubs/external_publications/EP69071.html

8. Whaley CM, Zhao X. The effects of physician vertical integration on referral patterns, patient welfare, and market dynamics. *J Public Econ*. 2024;238:105175. doi:10.1016/j.jpubeco.2024.105175

9. Wang Y, Meiselbach M, Anderson GF, Bai G. Hospital Pricing Information Consistent Between Transparency-In-Coverage Data And Other Commercial Data Sources. doi:10.1377/forefront.20231108.269718

10. Henderson M, Mouslim M. Cross-Validation of Insurer and Hospital Price Transparency Data. 2024;30:e247-e250.

11. Oakes AH, Ikard M, Patton C, et al. Understanding Variation in Negotiated Rates Using Novel Health Plan Price Transparency Data. *JAMA Health Forum*. 2024;5(9):e243020. doi:10.1001/jamahealthforum.2024.3020

© 2025 Singh Y et al. *JAMA Health Forum*.

eTable 1. Number of Observations Across Ownership Type, by CPT Code

CPT	Hospital	Office	PE	Total
99202	1,055,605	2,554,197	12,205	3,622,007
99203	3,718,818	7,711,001	53,028	11,482,847
99204	3,551,491	5,389,539	44,863	8,985,893
99205	445,147	609,008	3,090	1,057,245
99211	2,445,881	5,355,219	44,087	7,845,187
99212	2,587,913	5,360,733	42,271	7,990,917
99213	29,170,196	55,164,299	423,962	84,758,457
99214	37,442,225	57,139,252	659,476	95,240,953
99215	2,471,179	3,147,394	29,750	5,648,323
**Total**	**82,888,457**	**142,430,647**	**1,312,732**	**226,631,836**

Notes/Sources: Authors' calculation of Pitchbook, IQVIA SK&A, and TiC data.

© 2025 Singh Y et al. *JAMA Health Forum*.

eFigure 1. Share of Physicians in PE-Acquired Practices, 2009-2022

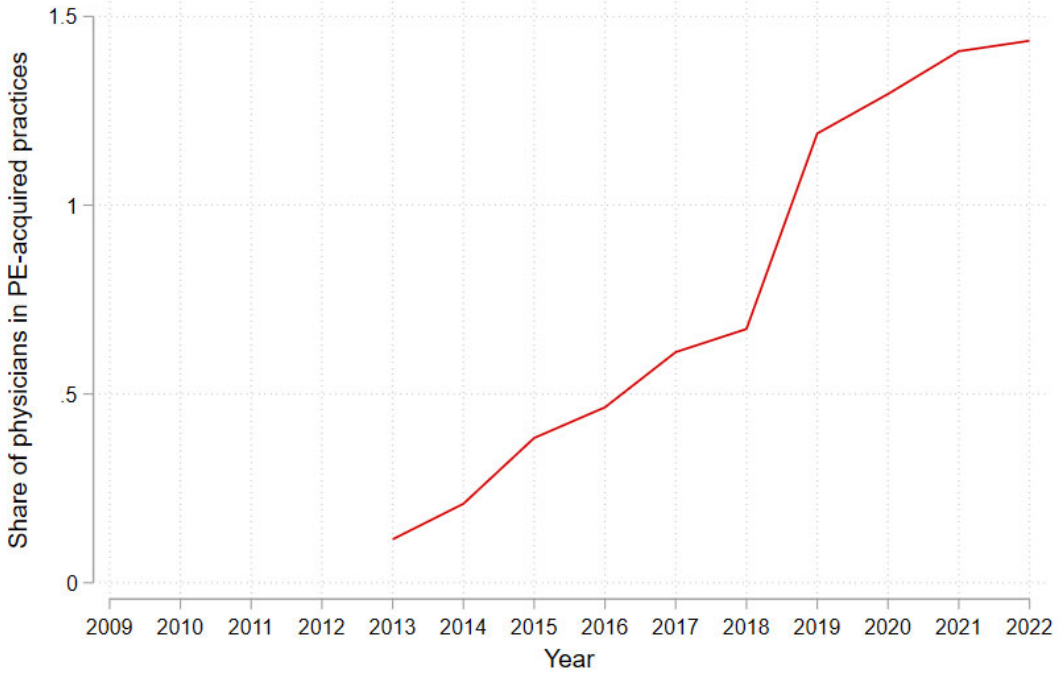

Notes/Sources: Authors' calculation of data from Pitchbook and IQVIA SK&A. Figure depicts the share of all primary care physicians affiliated with PE-acquired practices. The share of PE-acquired physicians was less than 0.1 percent before 2013 and is excluded from the figure.

© 2025 Singh Y et al. *JAMA Health Forum*.

eFigure 2. Unadjusted Prices for Office Visits Across Ownership Type, by CPT Code

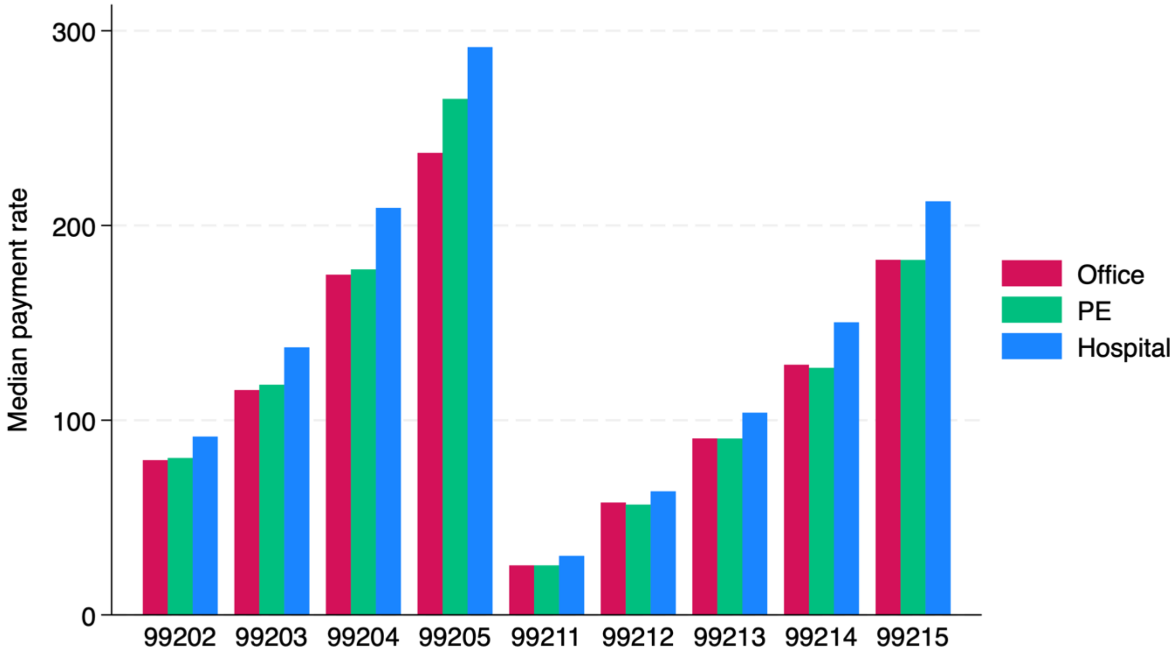

Notes/Sources: Authors' analysis of Pitchbook, IQVIA SK&A, and TiC data. This figure summarizes the median payment rate across 226.6 million reported prices for office visits with primary care physicians across ownership settings. Data on CPT 99201 was unavailable.

© 2025 Singh Y et al. *JAMA Health Forum*.

eFigure 3. Unadjusted Prices for Office Visits by Ownership Type, by Insurer

(a) United HealthCare

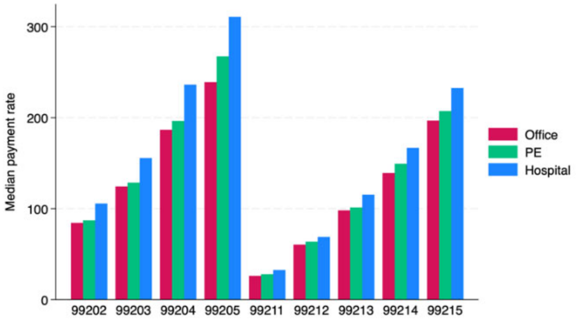

(b) Blue Cross Blue Shield

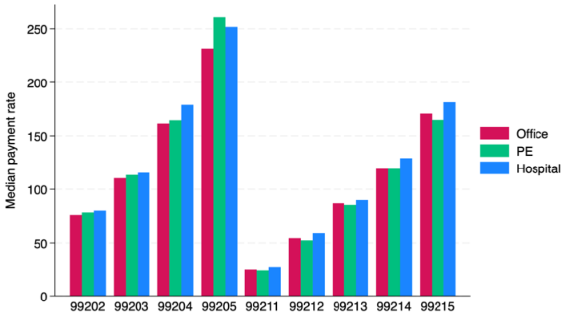

(c) Aetna

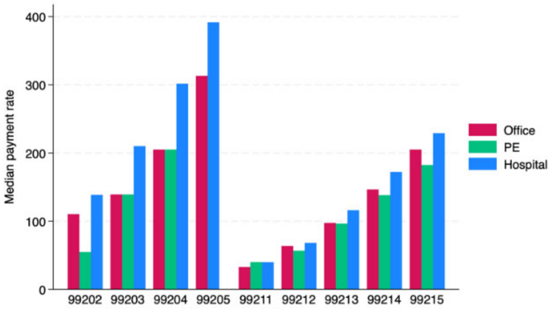

(d) Cigna

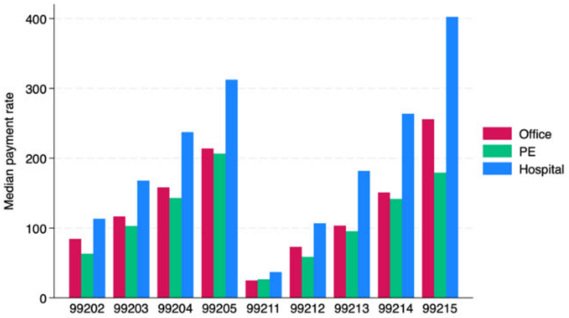

Notes/Sources: Authors' analysis of Pitchbook, IQVIA SK&A, and TiC data. This figure summarizes the median payment rate for office visits across ownership types for a given insurer. Data on CPT 99201 was unavailable.

© 2025 Singh Y et al. *JAMA Health Forum*.

eFigure 4. Geographic Variation in Prices for Office Visits With Hospital-Affiliated Primary Physicians Relative to Other Outpatient Settings

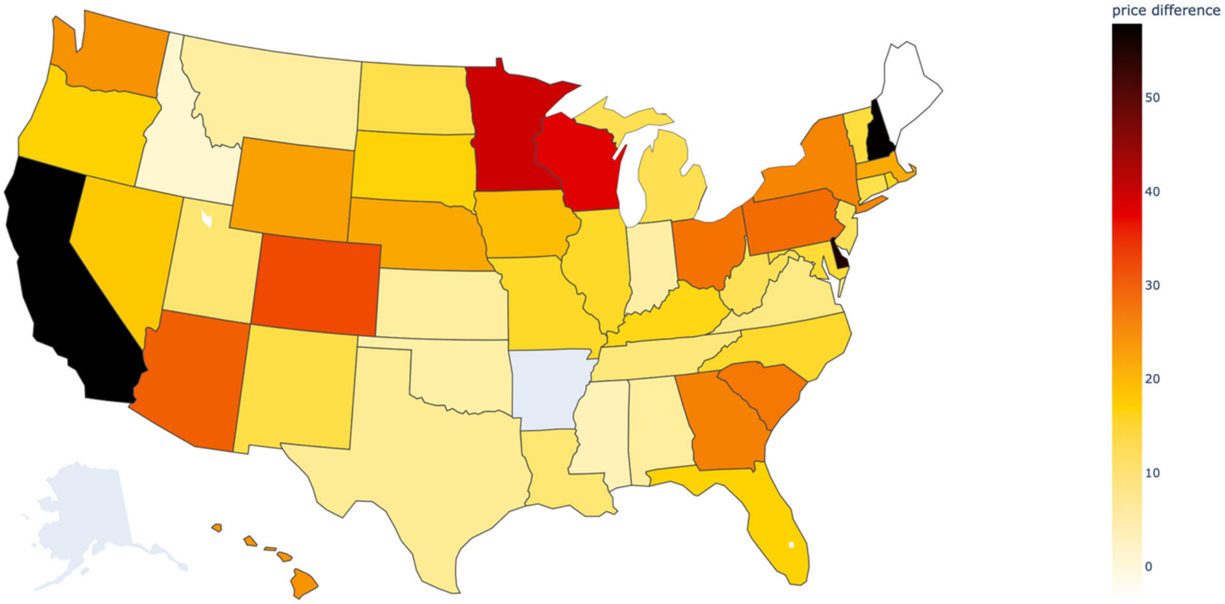

Notes: This map summarizes regression coefficients obtained from a fixed effects regression with negotiated price as the dependent variable and binary indicators for health system ownership interacted with state as independent variables. The reference category is all primary care practices that are not affiliated with health systems. Regressions include fixed effects for state, insurer, and service (CPT) code. Standard errors are clustered at the level of the state.

© 2025 Singh Y et al. *JAMA Health Forum*.

eFigure 5. Ranking of States With the Highest Price Differential Comparing Hospital-Affiliated Prices to All Other Prices

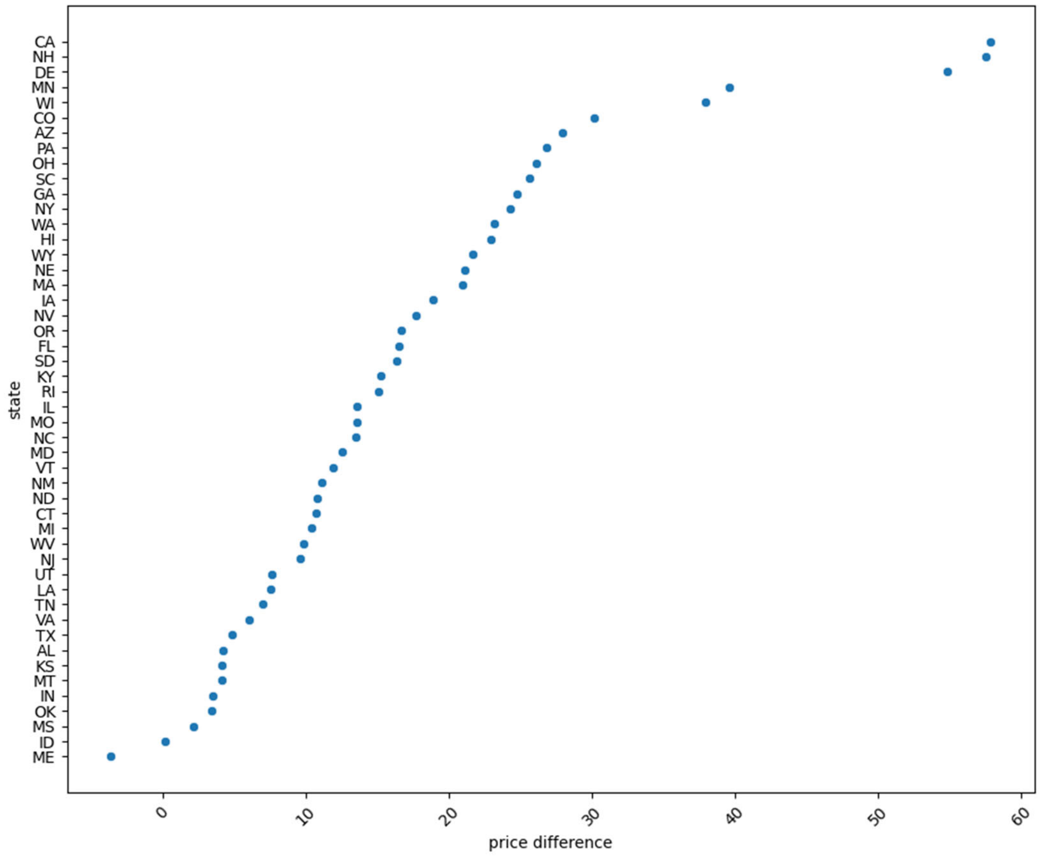

Notes: This plot summarizes regression coefficients obtained from a fixed effects regression with negotiated price as the dependent variable and binary indicators for health system ownership interacted with state as independent variables. The reference category is all primary care practices that are not affiliated with health systems. Regressions include fixed effects for state, insurer, and service (CPT) code. Standard errors are clustered at the level of the state.

© 2025 Singh Y et al. *JAMA Health Forum*.

eTable 2. Adjusted Variation in Commercial Prices for Office Visits by Ownership Type (Dollars, Alternate Data Source to Identify Hospital-Affiliated Physicians)

	Coefficient	Std. error	Lower confidence Interval	Upper Confidence Interval	P-value
**Ownership [Reference: Independent Office]**					
PE	9.847	1.966	5.994	13.700	< 0.01
Hospital	19.526	0.411	18.721	20.331	< 0.01
**Service code**					
New patient office visit, 30-44 minutes (CPT 99203)	40.187	0.912	38.399	41.975	< 0.01
New patient office visit, 45-59 minutes (CPT 99204)	109.430	0.968	107.533	111.328	< 0.01
New patient office visit, Greater than 60 minutes (CPT 99205)	182.354	1.389	179.631	185.076	< 0.01
Established patient office or other outpatient visit (CPT 99211)	-64.194	1.886	-67.892	-60.497	< 0.01
Established patient office or other outpatient visit, 10-19 minutes (CPT 99212)	-32.068	1.026	-34.079	-30.056	< 0.01
Established patient office or other outpatient visit, 20-29 minutes (CPT 99213)	6.530	0.837	4.891	8.170	< 0.01
Established patient office or other outpatient visit, 30-39 minutes (CPT 99214)	52.224	0.848	50.562	53.885	< 0.01
Established patient office or other outpatient visit, greater than 40 minutes (CPT 99215)	114.728	1.058	112.654	116.802	< 0.01
**Constant**	88.530	0.864	86.836	90.224	< 0.01

Notes: Table summarizes regression coefficients obtained from a fixed effects regression with physician professional fees as the dependent variable and binary indicators for ownership type (hospital-affiliated or PE-affiliated) as independent variables. The reference category is primary care physician practices under independent ownership. Regressions include fixed effects for service (CPT) code, and insurer-state (absorbed) and are weighted by the total commercial service volume for a given NPI. Standard errors are clustered at the level of the physician. Hospital-affiliation is identified using the Medicare Data on Provider Practice and Specialty (MD-PPAS), the Medicare Provider Enrollment, Chain, and Ownership System (PECOS), and IRS Form 990 tax forms to measure hospital-affiliated PCPs

© 2025 Singh Y et al. *JAMA Health Forum*.

eTable 3. Adjusted Variation in Commercial Prices for Office Visits by Ownership Type (Log-Transformed, Alternate Data Source to Identify Hospital-Affiliated Physicians)

	**Coefficient**	**Std. error**	**Lower confidence Interval**	**Upper Confidence Interval**	**P-value**
**Ownership [Reference: Independent Office]**					
PE	0.075	0.015	0.046	0.103	0.015
Hospital	0.127	0.003	0.122	0.132	< 0.01
**Service code**					
New patient office visit, 30-44 minutes (CPT 99203)	0.365	0.009	0.347	0.383	< 0.01
New patient office visit, 45-59 minutes (CPT 99204)	0.781	0.009	0.763	0.799	< 0.01
New patient office visit, Greater than 60 minutes (CPT 99205)	1.048	0.010	1.029	1.066	0.01
Established patient office or other outpatient visit (CPT 99211)	-1.150	0.015	-1.179	-1.121	0.015
Established patient office or other outpatient visit, 10-19 minutes (CPT 99212)	-0.380	0.011	-0.401	-0.358	0.011
Established patient office or other outpatient visit, 20-29 minutes (CPT 99213)	0.079	0.009	0.061	0.096	< 0.01
Established patient office or other outpatient visit, 30-39 minutes (CPT 99214)	0.450	0.009	0.433	0.468	< 0.01
Established patient office or other outpatient visit, greater than 40 minutes (CPT 99215)	0.785	0.009	0.767	0.803	< 0.01
**Constant**	4.454	0.009	4.436	4.472	< 0.01

Notes: Table summarizes regression coefficients obtained from a fixed effects regression with log-transformed physician professional fees as the dependent variable and binary indicators for ownership type (hospital-affiliated or PE-affiliated) as independent variables. The reference category is primary care physician practices under independent ownership. Regressions include fixed effects for service (CPT) code, and insurer-state (absorbed) and are weighted by the total commercial service volume for a given NPI. Standard errors are clustered at the level of the physician. Hospital-affiliation is identified using the Medicare Data on Provider Practice and Specialty (MD-PPAS), the Medicare Provider Enrollment, Chain, and Ownership System (PECOS), and IRS Form 990 tax forms to measure hospital-affiliated PCPs

© 2025 Singh Y et al. *JAMA Health Forum*.

eTable 4. Adjusted Variation in Commercial Prices for Office Visits by Ownership Type (Dollars, No Regression Weights)

	Coefficient	Std. error	Lower confidence Interval	Upper Confidence Interval	p-value
**Ownership [Reference: Independent Office]**					
PE	8.40	3.0606	2.24	14.55	< 0.01
Hospital	18.28	4.66	8.92	27.64	< 0.01
**Insurer**					
BCBS	-12.86	9.25	-31.46	5.73	0.17
Cigna	3.97	15.65	-27.49	35.45	0.80
UHC	6.32	10.21	-14.19	26.84	0.53
**Service code**					
99203	46.53	2.88	40.74	52.33	< 0.01
99204	120.38	6.83	106.65	134.10	< 0.01
99205	192.82	8.47	175.79	209.85	< 0.01
99211	-67.79	3.24	-74.31	-61.27	< 0.01
99212	-33.21	1.42	-36.07	-30.35	< 0.01
99213	9.91	1.73	6.44	13.38	< 0.01
99214	58.54	4.00	50.51	66.58	< 0.01
99215	122.40	7.76	106.80	138.01	< 0.01
**Constant**	91.95	13.12	65.57	118.33	< 0.01

Notes: Table summarizes regression coefficients obtained from a fixed effects regression with price as the dependent variable and binary indicators for ownership type (health system affiliated or PE-acquired) as independent variables. The reference category is primary care physician practices under independent ownership. Regressions include fixed effects for states (absorbed), insurer, and service (CPT) code. Standard errors are clustered at the level of the state.

© 2025 Singh Y et al. *JAMA Health Forum*.

eTable 5. Adjusted Variation in Commercial Prices for Office Visits by Ownership Type (Log-Transformed, No Regression Weights)

	Coefficient	Std. error	Lower confidence Interval	Upper Confidence Interval	p-value
**Ownership**					
PE	0.054403	0.0214525	0.0112698	0.0975362	0.015
Hospital	0.1092392	0.0229329	0.0631295	0.155349	< 0.01
**Insurer**					
BCBS	-0.1116696	0.0551088	-0.2224733	-0.0008659	0.048
Cigna	-0.0476349	0.0798463	-0.2081767	0.1129069	0.554
UHC	0.003361	0.0541183	-0.1054512	0.1121732	0.951
**Service code**					
99203	0.3901454	0.0056428	0.3787997	0.401491	< 0.01
99204	0.8044744	0.0090849	0.7862081	0.8227408	< 0.01
99205	1.07172	0.0077847	1.056068	1.087372	< 0.01
99211	-1.158731	0.0099321	-1.178701	-1.138762	< 0.01
99212	-0.3808452	0.0125231	-0.4060246	-0.3556658	< 0.01
99213	0.1087267	0.011673	0.0852565	0.1321968	< 0.01
99214	0.4713181	0.0099055	0.4514017	0.4912344	< 0.01
99215	0.8052135	0.0120197	0.7810462	0.8293808	< 0.01
**constant**	4.523329	0.0641147	4.394418	4.65224	< 0.01

Notes: Table summarizes regression coefficients obtained from a fixed effects regression with log-adjusted price as the dependent variable and binary indicators for ownership type (health system or PE-acquired) as independent variables. The reference category is primary care physicians under independent ownership. Regressions include fixed effects for states (absorbed), insurer, and service (CPT) code. Standard errors are clustered at the level of the state.

© 2025 Singh Y et al. *JAMA Health Forum*.

eTable 6. Adjusted Variation in Commercial Prices for Office Visits by Ownership Type (Log-Transformed, Regression Weights Defined as Total Commercial Volume for a Provider)

	Coefficient	Std. error	Lower confidence Interval	Upper Confidence Interval	P-value
**Ownership [Reference: Independent Office]**					
PE	0.075	0.014	0.046	0.103	0.015
Hospital	0.102	0.003	0.097	0.108	< 0.01
**Service code**					
New patient office visit, 30-44 minutes (CPT 99203)	0.367	0.009	0.350	0.384	< 0.01
New patient office visit, 45-59 minutes (CPT 99204)	0.786	0.009	0.768	0.803	< 0.01
New patient office visit, Greater than 60 minutes (CPT 99205)	1.053	0.009	1.035	1.071	< 0.01
Established patient office or other outpatient visit (CPT 99211)	-1.151	0.013	-1.176	-1.125	< 0.01
Established patient office or other outpatient visit, 10-19 minutes (CPT 99212)	-0.380	0.010	-0.400	-0.360	< 0.01
Established patient office or other outpatient visit, 20-29 minutes (CPT 99213)	0.083	0.009	0.066	0.099	< 0.01
Established patient office or other outpatient visit, 30-39 minutes (CPT 99214)	0.455	0.009	0.438	0.472	< 0.01
Established patient office or other outpatient visit, greater than 40 minutes (CPT 99215)	0.792	0.009	0.775	0.810	< 0.01
**Constant**	4.449	0.009	4.432	4.467	< 0.01

Notes: Table summarizes regression coefficients obtained from a fixed effects regression with log-transformed physician professional fees as the dependent variable and binary indicators for ownership type (hospital-affiliated or PE-affiliated) as independent variables. The reference category is primary care physician practices under independent ownership. Regressions include fixed effects for service (CPT) code, and insurer-state (absorbed) and are weighted by the total commercial service volume for a given NPI. Standard errors are clustered at the level of the physician.

© 2025 Singh Y et al. *JAMA Health Forum*.

eTable 7. Adjusted Variation in Commercial Prices for Office Visits by Ownership Type (Rate, Regression Weights Defined as Insurer Share)

	**Coefficient**	**Std. error**	**Lower confidence Interval**	**Upper Confidence Interval**	**p-value**
**Ownership**					
PE	6.723925	4.128293	-1.576564	15.02441	0.11
Hospital	16.11631	3.422949	9.234012	22.99861	< 0.01
**Insurer**					
BCBS	-17.88622	8.827976	-35.63606	-0.1363844	0.048
Cigna	-11.65263	14.03467	-39.87123	16.56596	0.41
UHC	-1.415513	9.314385	-20.14334	17.31231	0.88
**Service code**					
99203	47.23702	1.731086	43.75643	50.7176	< 0.01
99204	122.9491	3.742291	115.4247	130.4735	< 0.01
99205	193.4753	6.026088	181.359	205.5916	< 0.01
99211	-66.894	2.680028	-72.28256	-61.50545	< 0.01
99212	-33.24112	1.359751	-35.97508	-30.50716	< 0.01
99213	10.83508	1.544978	7.728697	13.94147	< 0.01
99214	58.49912	2.302956	53.86872	63.12952	< 0.01
99215	122.7704	4.346957	114.0303	131.5106	< 0.01
**constant**	99.25251	10.21187	78.72017	119.7849	< 0.01

Notes: Table summarizes regression coefficients obtained from a fixed effects regression with price as the dependent variable and binary indicators for ownership type (health system or PE-acquired) as independent variables. The reference category is primary care physicians under independent ownership. Regressions include fixed effects for states (absorbed), insurer, and service (CPT) code. Standard errors are clustered at the level of the state. Regression weights are defined as the insurer's share of commercial service volume.

© 2025 Singh Y et al. *JAMA Health Forum*.

eTable 8. Adjusted Variation in Commercial Prices for Office Visits by Ownership Type (Log-Transformed, Regression Weights Defined as Insurer Share)

	Coefficient	Std. error	Lower confidence Interval	Upper Confidence Interval	p-value
**Ownership**					
PE	0.0453276	0.0300226	-0.0150368	0.105692	0.138
Hospital	0.097931	0.0183987	0.060938	0.1349241	< 0.01
**Insurer**					
BCBS	-0.1235602	0.0540111	-0.2321567	-0.0149637	0.027
Cigna	-0.1211618	0.0762452	-0.2744631	0.0321395	0.119
UHC	-0.0317685	0.0527085	-0.137746	0.074209	0.55
**Service code**					
99203	0.3977276	0.0065388	0.3845805	0.4108747	< 0.01
99204	0.8213929	0.0083107	0.804683	0.8381028	< 0.01
99205	1.094278	0.0145013	1.065121	1.123435	< 0.01
99211	-1.141321	0.0229404	-1.187446	-1.095196	< 0.01
99212	-0.3918433	0.0107648	-0.4134874	-0.3701991	< 0.01
99213	0.120964	0.0143201	0.0921714	0.1497566	< 0.01
99214	0.477232	0.0129937	0.4511065	0.5033576	< 0.01
99215	0.8164634	0.014907	0.7864908	0.846436	< 0.01
**constant**	4.554958	0.0563688	4.441621	4.668295	< 0.01

Notes: Table summarizes regression coefficients obtained from a fixed effects regression with log-price as the dependent variable and binary indicators for ownership type (health system or PE-acquired) as independent variables. The reference category is primary care physicians under independent ownership. Regressions include fixed effects for states (absorbed), insurer, and service (CPT) code. Standard errors are clustered at the level of the state. Regression weights are defined as the insurer's share of commercial service volume.

© 2025 Singh Y et al. *JAMA Health Forum*.

eTable 9. Adjusted Variation in Commercial Prices for Office Visits by Ownership Type (Dollars and Log-Transformed, Select CPT Codes)

	Coefficient	Standard Error	p-value	Lower Confidence Interval	Upper Confidence Interval
**Outcome: Physician professional fees (CPT 99214)**					
pe	12.39457	2.361083	<0.01	7.766873	17.02226
hospital	17.08342	0.4394146	<0.01	16.22217	17.94467
Constant	139.4559	0.2695113	<0.01	138.9277	139.9842
**Outcome: Log-transformed physician professional fees (CPT 99214)**					
pe	0.082097	0.0155975	<0.01	0.0515261	0.1126679
hospital	0.1042314	0.0024532	<0.01	0.0994231	0.1090397
Constant	4.898316	0.0015975	<0.01	4.895184	4.901447
**Outcome: Physician professional fees (CPT 99213)**					
pe	8.637594	1.61481	<0.01	5.472587	11.8026
hospital	10.42387	0.4047484	<0.01	9.630567	11.21717
Constant	97.3295	0.2304066	<0.01	96.87791	97.7811
**Outcome: Log-transformed physician professional fees (CPT 99213)**					
pe	0.0809578	0.0147484	<0.01	0.0520511	0.1098645
hospital	0.0909994	0.003302	<0.01	0.0845275	0.0974713
Constant	4.538458	0.0018589	<0.01	4.534814	4.542101

Notes: Table summarizes regression coefficients obtained from a fixed effects regression with dollars and log-price as the dependent variable and binary indicators for ownership type (health system or PE-acquired) as independent variables. The reference category is primary care physicians under independent ownership. Regressions include fixed effects for states (absorbed) and insurer-state. Standard errors are clustered at the level of the physician. Regression weights are defined as (confirm)

© 2025 Singh Y et al. *JAMA Health Forum*.

eFigure 6. Proportion of Low vs High Complexity Visits Across Ownership Type

(a) New patient visits (CPTs 99202-99205)

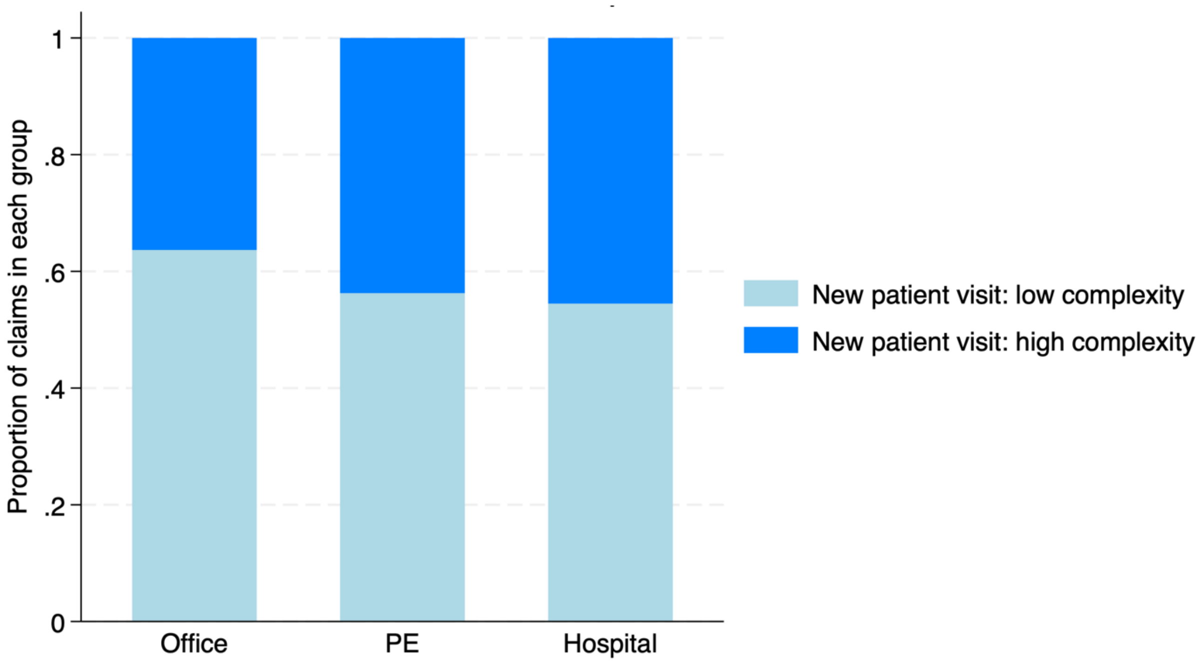

(b) Established patient visit (CPTs 99211-99215)

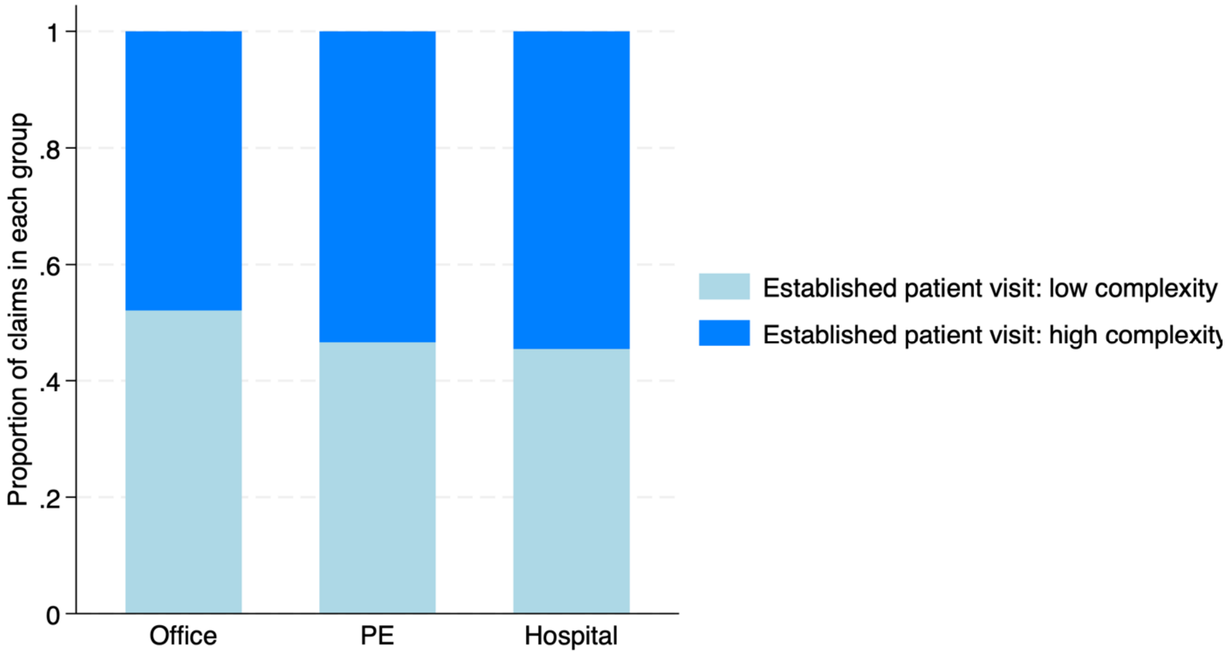

Notes/Sources: Authors' analysis of Pitchbook, IQVIA SK&A, and TiC data. This figure summarizes the proportion of office visit claims that are billed as low vs high complexity by ownership type. For new patient visits (CPTs 99202-99205), low complexity visits include CPTs 99202 and 99203 and for established patient visits (CPTs 99211-99215), low complexity visits include CPTs 99211-99213.

© 2025 Singh Y et al. *JAMA Health Forum*.

eTable 10. Changes in Health Care Spending Under Different Assumptions

	Health System-Affiliated		Private Equity-Affiliated	
	**Spending**	**Decrease in spending (%)**	**Spending**	**Decrease in spending (%)**
**Actual**	10,895,326,641		147,021,977	
**(A) Using median price at independent offices**	9,353,190,230	14%	147,760,670	--
**(B) Using median price and service complexity at independent offices**	9,040,655,594	17%	143,179,866	3%

Notes: Actual spending is calculated as the sum of the product of mean negotiated price and service volume for each CPT. In counterfactual (A) we calculate health care spending using median negotiated prices at independent settings and total service volume at the health system and PE-affiliated practices respectively. In counterfactual (B) we calculate health care spending using median negotiated prices and service-mix at independent settings.

© 2025 Singh Y et al. *JAMA Health Forum*.
