# Supplementary material for: Growth of Private Equity and Hospital Consolidation in Primary Care and Price Implications
Source: JAMA Health Forum. 2025 Jan 17;6(1):e244935. doi: 10.1001/jamahealthforum.2024.4935 (PMC11742525; doi:10.1001/jamahealthforum.2024.4935)
Supplement: Supplement 2. — Data Sharing Statement [file jamahealthforum-e244935-s002.pdf]

Data Sharing Statement

Singh. Growth of Private Equity and Hospital Consolidation in Primary Care and Price Implications. *JAMA Health Forum*. Published January 17, 2025.
doi:10.1001/jamahealthforum.2024.4935

Data
Data available: No
